# Self-collected vaginal sampling for the detection of genital human papillomavirus (HPV) using *care*HPV among Ghanaian women

**DOI:** 10.1186/s12905-017-0448-1

**Published:** 2017-09-26

**Authors:** Dorcas Obiri-Yeboah, Yaw Adu-Sarkodie, Florencia Djigma, Anna Hayfron-Benjamin, Latif Abdul, Jacques Simpore, Philippe Mayaud

**Affiliations:** 1 Department of Microbiology and Immunology, School of Medical Sciences, University of Cape Coast, Cape Coast, Ghana; 2 Department of Clinical Microbiology, School of Medical Sciences, Kwame Nkrumah University of Science and Technology, Kumasi, Ghana; 3 Laboratory of Molecular Biology and Genetics (LABIOGENE), University of Ouagadougou, Ouagadougou, Burkina Faso; 4 Department of Maternal and Child Health, School of Nursing, University of Cape Coast, Cape Coast, Ghana; 5 Department of Clinical Research, Faculty of Infectious and Tropical Diseases, London School of Hygiene and Tropical Medicine, London, UK

**Keywords:** Human papillomavirus (HPV), Self-collection, Clinician-collection, careHPV, Ghana, Cervical cancer

## Abstract

**Background:**

Detection of genital HPV DNA is recommended as an important strategy for modern cervical cancer screening. Challenges include access to services, the reliance on cervical samples taken by clinicians, and patient’s preference regarding provider gender. The objective of this research was to determine the acceptability, feasibility and performance of alternative self-collected vaginal samples for HPV detection among Ghanaian women.

**Methods:**

A comparative frequency-matched study was conducted in a systematic (1:5) sample of women attending HIV and outpatient clinics in the Cape Coast Teaching Hospital, Ghana. Participants were instructed on self-collection (SC) of vaginal samples using the *care*HPV brush and a clinician-collected (CC) cervical sample was obtained using a similar brush. Paired specimens were tested for HPV DNA (14 high-risk types) by *care*HPV assay (Qiagen) and by HPV genotyping (Anyplex II, Seegene).

**Results:**

Overall, 194 women of mean age 44.1 years (SD ± 11.3) were enrolled and 191 paired SC and CC results were analysed. The overall HPV detection concordance was 94.2% (95%CI: 89.9–97.1), Kappa value of 0.88 (*p* < 0.0001), showing excellent agreement. This agreement was similar between HIV positive (93.8%) and negative (94.7%) women. Sensitivity and specificity of SC compared to CC were 92.6% (95%CI: 85.3–97.0) and 95.9% (95%CI: 89.8–98.8) respectively. The highest sensitivity was among HIV positive women (95.7%, 95%CI: 88.0–99.1) and highest specificity among HIV negative women (98.6%, 95%CI: 92.4–100). Overall, 76.3% women found SC very easy/easy to obtain, 57.7% preferred SC to CC and 61.9% felt SC would increase their likelihood to access cervical cancer screening.

**Conclusions:**

The feasibility, acceptability and performance of SC using *care*HPV support the use of this alternative form of HPV screening among Ghanaian women. This could be a potential new affordable strategy to improve uptake of the national cervical cancer screening program.

## Background

In Ghana, as in many parts of sub-Saharan Africa, cervical cancer is the commonest cancer and the leading cause of cancer-related mortality among women, with an estimated 3052 new case and 1556 deaths in Ghana annually [1]. The human papillomavirus (HPV) has been aetiologically linked to the development of cervical cancer (and other genital cancers) with persistence of infection with high-risk (hr) oncogenic HPV genotypes in the transformation zone of the cervix as the main contributing factor [2]. The prevalence of HPV is high in West Africa with an average of 20% among women without any abnormal cytology compared to an average 12% worldwide [3, 4]. Information on HPV prevalence and genotype distribution among Ghanaian women is limited, but available data suggest a significant burden and contribution to cervical cancer [5, 6].

The diagnosis of cervical cancer relies on the detection of precursor lesions using conventional cytology with pap smear, visual inspection using acetic acid or Lugol’s iodine (VIA/VILI) or colposcopy with histological confirmation, with varying performance characteristics for all of these tests and several limitations for implementation in low-resource settings [7].

Detection of hrHPV types has important prognostic value for the detection of high-grade cervical squamous intraepithelial lesions (HSIL) or neoplasia (CIN) [8]. Diagnosing HPV infection and its associated clinical conditions in Ghana is very challenging. The challenges include, but are not limited to, low levels of awareness in the general population [9] and the reliance on cervical samples taken by clinicians for diagnosis. The problem of clinician collection of cervical samples may limit acceptability and hence coverage of screening for various reasons. In industrialized countries, this could include the busy lifestyle of women who may not have the time to go to the clinic to have the sample taken, and the reluctance of some women to attend health facilities. Thus, any screening program which must be done in a clinic setting may not be suitable. In addition, the sample collection procedure requiring the passing of a vaginal speculum is uncomfortable and hence may act as a deterrent. The issue gets more complex in the diverse socio-cultural settings in Africa. Cultural beliefs frown upon another person aside the husband having access to the genital area of a female. A recent study in Ghana using a mixed methods approach confirmed that one barrier to cervical cancer screening mentioned by women was the cultural belief that it is inappropriate for another person to see a woman’s genital area [10].

Since highly sensitive molecular testing for HPV does not necessarily require exact cervical location of HPV replication (i.e. the squamo-columnar junction or transformation zone) to be collected, contrary to what cervical cytology requires, an additional potential benefit of HPV testing is the possible use of self-collected cervico-vaginal samples. It has been shown that self-collection might be preferred by women as it addresses issues affecting clinician sample collection [11–13]. Taylor et al. in their recent systematic review of self-sampling for sexually transmitted infections including HPV screening concluded that in addition to being acceptable to patients, this might also be a cost effective method [14]. Despite evidence of higher acceptability of self-collection, there is the need to evaluate the performance of such samples for HPV detection and to determine the views of women in a range of settings and backgrounds. This study sought to determine the performance of self-collected (SC) cervico-vaginal samples for HPV detection compared to clinician collection (CC) and to assess the preferred sampling method for women in the specific socio-cultural setting of Ghana.

## Methods

### Study subjects

Participants were recruited as part of a larger HPV and cervical cancer study conducted in the Cape Coast Teaching Hospital (CCTH), aiming at studying the epidemiology of, and diagnostic options for hrHPV and cervical lesions among HIV-1 seropositive compared with HIV-seronegative women. In the parent study, women attending the general medical outpatient and HIV clinics of CCTH were recruited. Every fifth woman aged ≥18 years was systematically selected from the list of daily attendants, starting with a randomly picked attendance number for the first woman. If a woman was deemed not eligible based on criteria for this study (currently menstruating, previous treatment of cervical cancer etc.), or she did not accept to be part of the study, the next available patient was offered her place, and every fifth woman whence, to a maximum of 10 women per clinic day was recruited. For the current evaluation of self-sampling, we aimed to have about half the sample size and participants were recruited on alternate days.

### Sample collection

To simulate home-based sampling, recruited participants at the clinic were instructed on how to obtain SC vaginal samples using the *care*HPV brush and to place it in the *care*HPV collection medium (Qiagen, Gaithersburg, MD). Participants then underwent a speculum exam during which clinicians collected a cervical sample using the *care*HPV brush and transport medium. After sample collection, participants filled a short questionnaire to assess their experience and views of SC compared with CC. The questionnaire was administered by the clinician who collected the sample. Four questions were asked, with two using a Lichter’s scale of possible responses (Table 2).

### HPV DNA detection

Paired SC and CC samples were tested using the *care*HPV assay (Qiagen, Gaithersburg, MD) following the manufacturer’s instructions. *care*HPV is a semi-rapid qualitative assay designed as a simplification of the Digene Hybrid Capture-II (HC2) test technology to detect the DNA of any of 14 hrHPV types (HPV16, 18, 31, 33, 35, 39, 45, 52, 56, 58, 59, 68, with HPV66 being an addition to the 13 hrHPV detected by HC2) [15–17]. The assay has been designed for use in resource-constrained settings with little need for refrigeration of samples prior to testing and using portable battery-operated reading machine. *care*HPV has been favourably evaluated in comparison to HC2 and other HPV detection assays for the detection of high-grade cervical lesions [18, 19].

In addition, HPV genotyping was done to verify type-specific concordance. An aliquot from each of the cervical and vaginal samples were tested using the Anyplex™ II HPV HR Detection assay (Seegene, South Korea) which detects 28 HPV types, including the same 14 hrHPV (16, 18, 31, 33, 35, 39, 45, 51, 52, 56, 58, 59, 66, 68) detected by *care*HPV. This assay has been validated against an international reference method [20].

### Sample size and statistical analysis

A Kappa calculation was performed to determine the sample size needed for this sub-study. A Kappa value of 0.75 or higher was taken to indicate excellent agreement. Assuming a ≤ 10% difference in levels of detection between SC vaginal samples versus comparator standard CC cervical sampling, it was estimated that a total of 150 paired SC and CC samples were needed.

The proportion of overall *care*HPV results agreement between paired SC and CC samples was calculated using the Kappa statistic. Sensitivity and specificity of SC compared to CC as the gold standard were also calculated with their 95% confidence intervals (CI). A genotype level analysis of concordance was also conducted. To determine the acceptability of SC and CC, the proportions of responses to questions asked concerning the experience of participants was calculated.

### Ethics

Ethical approval for the parent and this sub-study was obtained from the Committee on Human Research Publications and Ethics of the School of Medical Sciences, Kwame Nkrumah University of Science and Technology (KNUST). Study participants were recruited to this sub-study after obtaining additional signed written informed consent.

## Results

Between July 2014 and August 2015, a total of 333 women were recruited to the parent study (mean age 44.1 years, standard deviation [SD] ±11.2 years, 48.9% HIV-positive), of whom 195 eligible women were invited to be part of this sub-study. In all, 194 consenting women (mean age 44.1 years, SD ±11.3 years, 50% HIV-positive) provided paired SC and CC samples. *care*HPV invalid results rate after retesting was 1.5% (*n* = 3) and results from these women were excluded from the analysis.

### Concordance between SC and CC: *care*HPV results and genotypes

The overall *care*HPV concordance between the remaining 191 paired samples was 94.2% (Kappa = 0.88, *p* ≤ 0.0001) showing excellent agreement with similar high agreement among HIV seropositive (Kappa = 0.84, *p* < 0.0001) and seronegative (Kappa = 0.86, p < 0.0001) women (Table 1). The sensitivity of SC compared to CC was 92.6% (95%CI: 85.3–97.0%) and specificity was 95.9% (95%CI: 89.8–98.9%). The sensitivity was higher among HIV-seropositive women (95.7%, 95%CI: 88.0–99.1) and specificity was higher among HIV-seronegative women (98.6%, 95%CI: 92.4–100) (Table 1). The ability to take a good SC sample, defined as providing SC sample which produced the same result as the CC sample, was not affected by age (*p* = 0.570), educational level (*p* = 0.482), or HIV status (*p* = 1.000).

**Table 1:** Performance characteristics of SC samples compared with CC for 191 women and then by HIV status

Parameter	Overall (*N* = 191), % (95%CI)	HIV positives (*N* = 96), % (95%CI)	HIV negatives (N = 95), % (95%CI)
Concordance	94.2 (89.9–97.1)	93.8 (81.7–94.9)	94.7 (82.8–95.6)
Kappa value	0.88 (0.82–0.95)	0.84 (0.72–0.96)	0.88 (0.73–0.98)
Kappa *p* value	<0.0001	<0.0001	<0.0001
Sensitivity	92.6 (85.3–97.0)	95.7 (88.0–99.1)	83.3 (62.6–95.3)
Specificity	95.9 (89.8–98.9)	88.5 (69.8–97.6)	98.6 (92.4–100)

Among HIV positive women, for twelve (12) of the hrHPV types detected by *care*HPV, the positive agreement between SC and CC samples was 100%. For HIV negative women, the positive agreement between SC and CC samples was 100% for ten (10) hrHPV genotypes. The lowest positive agreement rates (50%) was among HIV negative women for HPV39 (Table 2).

**Table 2:** Positive agreement between clinician-collected (CC) and self-collected (SC) *care*HPV samples for the detection of 14 high-risk HPV genotypes among 191 women in Cape Coast Teaching Hospital, Ghana

hrHPV genotype	HIV POSITIVE (*N* = 96)			HIV NEGATIVE (*N* = 95)		
	CC	SC, n (%)	^a^Positive Agreement, %	CC	SC, n (%)	^a^Positive Agreement, %
16	12 (12.5)	12 (12.5)	100	1 (1.1)	1 (1.1)	100
18	12 (12.5)	12 (12.5)	100	2 (2.1)	2 (2.1)	100
31	12 (12.5)	12 (12.5)	100	0 (0.0)	0 (0.0)	100
33	7 (7.3)	7 (7.3)	100	5 (5.3)	5 (5.3)	100
35	13 (13.5)	13 (13.5)	100	4 (4.2)	3 (3.2)	75.0
39	6 (6.3)	6 (6.3)	100	2 (2.1)	1 (1.1)	50.0
45	7 (7.3)	7 (7.3)	100	1 (1.1)	1 (1.1)	100
51	1 (1.0)	1 (1.0)	100	0 (0.0)	0 (0.0)	100
52	14 (14.5)	14 (14.5)	100	1 (1.1)	1 (1.1)	100
56	8 (8.3)	8 (8.3)	100	3 (3.1)	2 (2.1)	66.7
58	13 (13.5)	14 (14.5)	92.9	4 (4.2)	5 (5.3)	80.0
59	5 (5.2)	5 (5.2)	100	0 (0.0)	0 (0.0)	100
66	4 (4.2)	5 (5.2)	80.0	2 (2.1)	2 (2.1)	100
68	9 (9.4)	9 (9.4)	100	1 (1.1)	1 (1.1)	100

^a^The definition of “positive agreement” is proportion of individuals with concordant CC and SC samples test results

The performance of *care*HPV compared with genotyping for women with cytological endpoint is presented elsewhere [21]. All women were followed up with cytology tests and management by a gynecologist, as appropriate.

### Acceptability of self-collection

Responses showed that over three quarters (76.3%) of women felt it was easy or very easy to self-collect vaginal samples and 77.9% felt SC was very comfortable or somehow comfortable compared with CC. Given the option, the majority of women (57.7%) would prefer SC over CC and nearly two-thirds (61.9%) felt SC would increase their likelihood to access cervical cancer screening (Table 3).

**Table 3:** Acceptability of self-collection compared with clinician collection of samples for HPV screening among 194 women at Cape Coast Hospital, Ghana

Item	Parameter	Options	Grade	Responses *n* (%)
1	How easy was SC for you?	Very easy	1	36 (18.6)
		Easy	2	112 (57.7)
		Difficult	3	45 (23.2)
		Very Difficult	4	1 (0.5)
2	How comfortable was the process of SC compared with CC?	Very comfortable	1	31 (16.0)
		Somewhat comfortable	2	120 (61.9)
		Somewhat uncomfortable	3	43 (22.2)
		Very uncomfortable	4	0 (0.0)
3	If you had the option, will you prefer SC or CC (having experienced both)?	SC		112 (57.7)
		CC		76 (39.2)
		Not sure		6 (3.1)
4	Do you think SC method will make you more likely to have cervical cancer screening?	Yes		120 (61.9)
		No		22 (11.3)
		Not sure		52 (26.8)

*SC* self-collected*, CC* clinician-collected

## Discussion

The study found that nearly all women could obtain a self-collected vaginal sample giving valid HPV results, and that they felt the method was acceptable and easy to use. Furthermore, the agreement between self-collected and clinician-collected samples for *care*HPV was excellent. This was equally the case for HIV sero-positive women and sero-negative women. Not surprisingly, the sensitivity of SC was higher among HIV sero-positive women since they have a higher risk of HPV infection, whilst specificity was highest among HIV sero-negative women. At the genotype level, the lowest degree of concordance was 50% for HPV39 among HIV negative women, but for the majority of hrHPV genotypes concordance was 90–100% among both groups. This study supports self-collection of vaginal sample as an excellent alternative sampling method for hrHPV detection, as found by others [13, 22–24], which could improve uptake and coverage of cervical cancer screening programs in low-resource settings [12]. Self-sampling offers many advantages, such as the ability to choose the time and place of collection, which, coupled with the stability of *care*HPV in the field (no need of refrigeration) and its performance to detect hrHPV infection, makes it a relevant approach for community level screening. In such campaigns, women could obtain samples at their homes, which could be brought to, or collected by, community health workers who would forward them to the laboratory. Results could easily and quickly be communicated back to the women without having to come to the health facility. With such a setup, only women who need follow-up tests like cytology or colposcopy would be invited to health facilities. This would alleviate the costs and burden on health services. The Community-based Health Planning and Services (CHP) concept, which already exists in Ghana, makes this a potentially easily adoptable strategy. Based on this concept, another research team is currently working on evaluating self-collection at the community-level in the Volta Region of Ghana.

In this study SC samples were taken with the same brush as for CC sampling without the need to purchase additional devices specifically marketed for SC, including the Delphi® screener (Delphi Biosciences, The Netherlands) and the Evalyn® brush (Rovers Medical Devices, The Netherlands). Thus, by using *care*HPV SC came at no additional cost - an important consideration in a developing country like Ghana.

This study reported that three-quarters of women found SC to be more acceptable than CC. Having experienced both SC and CC, the participants could give a realistic account of their experience and determine their preference. Researches in other settings, including rural communities, have reported similar findings [11, 25]. Trope et al. in their community-based screening study comparing SC and CC using *care*HPV among 431 women in rural Thailand recorded over 90% acceptability with over 70% of respondents indicating a preference for SC [26]. Even if women came to health facilities for screening, the use of SC may still increase acceptability of testing by solving the cultural and other challenges associated with CC. Furthermore, SC would help reduce the workload of health workers by eliminating or reducing the need to take all samples for HPV testing. However, some women may experience difficulties in self-collecting swabs for physical reasons, such as arthritis. In these cases, clinician sampling is preferable.

This study had some limitations. First, it used a quantitative approach to determine acceptability of self-collection. A complementary qualitative methodology would have enabled a better exploration of the women’s experience and potential difficulties. Second, women were recruited in a clinic setting, which increases the chances of participation, compliance with methodology and a positive outcome, as noted by others [24]. A larger community-based study, currently ongoing in the Volta Region, is required to determine the true acceptability and validity of self-collection in the context of mass screening in Ghana. Despite these limitations, this study provides useful encouraging addition to the limited data in Ghana on the role of self-collection of genital samples for hrHPV detection.

## Conclusion

This study therefore concludes that, the feasibility, acceptability and performance of vaginal self-collection using the *care*HPV assay support it as a good alternative for hrHPV testing Ghana.

## Abbreviations

ART: Anti-retroviral therapy
CC: Clinician collected
CCTH: Cape Coast Teaching Hospital
CI: Confidence interval
HIV: Human immunodeficiency virus
HPV: Human papilloma virus
hrHPV: High-risk human papilloma virus
KNUST: Kwame Nkrumah University of Science and Technology
PLHIV: People Living with HIV
SC: Self-collected
SIL: Squamous intraepithelial lesion
SSA: Sub Saharan Africa
STI: Sexually transmitted infection
